# Synergistic Chemotherapy Drug Response Is a Genetic Trait in Lymphoblastoid Cell Lines

**DOI:** 10.3389/fgene.2019.00829

**Published:** 2019-10-15

**Authors:** Kyle R. Roell, Tammy M. Havener, David M. Reif, John Jack, Howard L. McLeod, Tim Wiltshire, Alison A. Motsinger-Reif

**Affiliations:** ^1^Department of Statistics, North Carolina State University, Raleigh, NC, United States; ^2^Bioinformatics Research Center, North Carolina State University, Raleigh, NC, United States; ^3^Pharmacotherapy and Experimental Therapeutics, University of North Carolina at Chapel Hill, Chapel Hill, NC, United States; ^4^The DeBartolo Family Personalized Medicine Institute, Moffitt Cancer Center, Tampa, FL, United States; ^5^Center for Pharmacogenomics and Individualized Therapy, University of North Carolina at Chapel Hill, Chapel Hill, NC, United States; ^6^Biostatistics and Computational Biology Branch, National Institute of Environmental Health Sciences, Durham, NC, United States

**Keywords:** synergy, heritability, chemotherapy, lymphoblastoid cell lines, linkage mapping

## Abstract

Lymphoblastoid cell lines (LCLs) are a highly successful model for evaluating the genetic etiology of cancer drug response, but applications using this model have typically focused on single drugs. Combination therapy is quite common in modern chemotherapy treatment since drugs often work synergistically, and it is an important progression in the use of the LCL model to expand work for drug combinations. In the present work, we demonstrate that synergy occurs and can be quantified in LCLs across a range of clinically important drug combinations. Lymphoblastoid cell lines have been commonly employed in association mapping in cancer pharmacogenomics, but it is so far untested as to whether synergistic effects have a genetic etiology. Here we use cell lines from extended pedigrees to demonstrate that there is a substantial heritable component to synergistic drug response. Additionally, we perform linkage mapping in these pedigrees to identify putative regions linked to this important phenotype. This demonstration supports the premise of expanding the use of the LCL model to perform association mapping for combination therapies.

## Introduction

The current paradigm in cancer treatment is moving toward combination chemotherapy—the use of more than one medication simultaneously (Greco et al., 1996; Foucquier and Guedj, 2015). Since chemotherapy drugs can affect cancer cells at different points in the cell cycle, using a combination of drugs increases the chance that all of the cancer cells are eliminated and that resistance is prevented. For example, one drug may disrupt DNA replication, while a second drug interferes with protein synthesis. Attacking the malignant cells through different mechanisms lessens the chance of a mutated cell line evolving to develop deadly drug resistance. Ultimately, combination therapy approaches are optimized for synergistic interactions—where the effect of the combination interacts in a nonadditive way for improved outcomes (Musgrove et al., 2011; Janku et al., 2014). Recent studies have demonstrated higher response rates with combinations of drugs compared to monotherapies, and the Food and Drug Administration has recently even approved a first-of-its-kind “Co-Pack” treatment ( A growing body of evidence supports the efficacy of combination therapy (Liu et al., 2014). For example, a recent study of solid cancers showed that in most clinical cases combination therapies are needed to avoid the evolution of resistance to targeted drugs (Bozic et al., 2013). Furthermore, they find that the simultaneous administration of multiple targeted drugs minimizes the chance of relapse when no single mutation confers cross-resistance to both drugs (Bozic et al., 2013).

While the population-level benefits of combination therapy are often clear, there are a number of downsides and risks that need to be taken into account at the individual patient level (Riechelmann et al., 2007). First, drug combinations can synergistically or additively create therapeutic benefits, but can equally produce accumulating toxicities and other adverse outcomes (Delbaldo et al., 2004). With combination therapy, it is not possible to know which drug is causing the side effect, making it difficult to know which drug’s dose should be adjusted. If the therapeutic agents have similar side effect profiles, the accumulation of side effects can create more severe clinical side effects and grade 3/4 toxicities. For example, Berdeja et al. (2015) demonstrated that combination therapy with panobinostat and carfilzomib in patients with multiple myeloma resulted in treatment-related heart failure (2%), and treatment-related death rose (2%). These concerns can greatly impact the well-being and overall prognosis of patients.

Obviously, being able to predict which patients will respond positively and which will respond negatively would be an ideal situation. Such precision/personalized medicine approaches are limited by a general lack of understanding of the etiology of drug synergy. Just as there is variability in patients’ response to single drugs, it is generally accepted that individual response to combination therapy can be variable, and the underlying reasons for this are not well understood. It has been well established in lymphoblastoid cell lines (LCLs) that dose response to single drug exposure has a genetic component; thus, an underlying genetic etiology of drug synergy could be a viable mechanism of interindividual variation in the synergistic response (Peters et al., 2011; Watson et al., 2011; Abdo et al., 2015).

However, in pharmacogenomics, there are limited systems in which this hypothesis can be tested. In fact, the challenge of establishing whether drug response traits in general are not only genetic in their etiology but also heritable is a particular in pharmacogenomics in general (Motsinger-Reif et al., 2013). One major reason for this is that family designs are needed for true heritability estimates, which is often impractical in a drug response experiment. Additionally, establishing synergy is further complicated by the need to first establish single drug response. This motivates the use of model systems to further research on synergistic interactions.

Lymphoblastoid cell lines are a highly successful model for dissecting the genetic etiology of cancer drug response, including estimating the heritability of drug response traits and performing both linkage and association mapping to identify putative regions and genes that explain variability in dose response (Watson et al., 2011; Moen et al., 2012; Wheeler et al., 2013; Niu et al., 2016; Jack et al., 2018). As reviewed here, the LCL model system has resulted in a number of translatable successes (Jack et al., 2014). This includes examples of findings that have been translated into clinical samples and those that have led to new insights into the biological mechanisms of drug response after a range of follow-up experiments to explore the genetic findings from the LCLs (Moen et al., 2012; Wheeler et al., 2013; Abdo et al., 2015).

A recent study demonstrated response to carboplatin, paclitaxel, and their combination in LCLs (Fridley et al., 2016). Although synergy was not directly quantified, the research found numerous loci associated with drug response both in the monotherapy and combination therapy experiments and suggested the need for follow-up experimentation and further evaluation of combination experiments in the model system (Fridley et al., 2016). In the current study, we explore the potential synergistic effects of important chemotherapy drug combinations in the LCL system using methods for synergy quantification. Additionally, we evaluate the response of the cell lines to an increasing concentration of both singular and combination drug exposures. We used a family-based pedigree structure to both quantify synergistic effects in the cell lines and estimate the heritable component of these effects. Additionally, we perform linkage analysis to map the regions of the genome that are responsible for the genetic component of the drug response traits. Such linkage results provide insight into the potential genetic etiology underlying the heritability. The present study also includes seven unique combinations of drugs, allowing us to potentially evaluate if synergy is a common occurrence in these cell lines.

## Materials and Methods

### Cell Lines

Lymphoblastoid cell lines were obtained from the Centre de’Etude du Polymorphism Humain (CEPH) pedigrees (CEPH Genotype Database) that are commercially available from the Coriell Cell Repositories (NJ, USA). These LCLs are collected from healthy human volunteers and are generally established from large, multigenerational families. Comprehensive and publicly available genotype data are easily accessible for these cell lines and were downloaded and quality controlled as described below. For the current study, 126 LCLs including 45 trios (a set of parents and single child) as well as lines consisting of multiple children and their parents were used. The cell lines used were from the following pedigrees: 35, 45, 1334, 1340, 1341, 1345, 1350, 1362, 1408, 1420, 1447, 1451, 1454, and 1459.

Fresh lymphoblastoid cells were received from Coriell in T25 flasks. They were further cultured in Roswell Park Memorial Institute medium 1640 containing 2 mM l-glutamine (Gibco, CA, USA) and 15% fetal bovine serum (Sigma-Aldrich Co, MO, USA; lot 107K8408) at 37°C, 5% CO_2_ in a NUAIRE autoflow infrared direct heat CO_2_ incubator (Labrepco, PA, USA). No antibiotics were used. Frozen aliquots of each cell line were stored in liquid nitrogen for use throughout the study. Individual cell lines were counted in a Z1 Coulter Particle Counter (Beckman Coulter, CA, USA) and plated at 4,000 cells/well (45 µL per well) in a 384-well plate containing the drugs (as noted in the “Anticancer Drugs” section) for the dose–response assays. One cell line was assayed per 384-well plate, and replicates were performed using a new frozen aliquot grown at a different time. The order of cell lines for the assays was randomized prior to conducting the assays. This was done to prevent any systematic bias by family or family structure.

### Anticancer Drugs

Literature searches were performed to identify combinations of anticancer drugs that demonstrated synergy in at least two distinct cancer cell line systems (Photiou et al., 1997; Zoli et al., 1999; Edelman et al., 2001; Budman and Calabro, 2002; Vogt et al., 2004; Nannizzi et al., 2010). The drugs and their combinations were also chosen with a view to the broad use of these drugs in clinical settings. For example, docetaxel is used to treat many types of cancer including breast, prostate, stomach, head and neck, and non–small cell lung cancer. Other drugs, like epirubicin, are used for a more limited number of cancer types, but are among the most commonly prescribed chemotherapeutics. As most of these examples were from solid tumor–derived cell lines, initial laboratory studies were conducted in five lymphoblastoid cell lines to confirm the cytotoxic concentration range for each individual drug, and that synergy was apparent in this cell system. The drugs and their mechanisms are shown in **Table 1**. The following drug combinations were used: docetaxel and vinorelbine, docetaxel and gemcitabine, docetaxel and epirubicin, oxaliplatin and 5-fluorouracil, paclitaxel and epirubicin, paclitaxel and vinorelbine, and paclitaxel and carboplatin. Drugs were obtained from LKT or Sigma, and solutions were made using either water or a combination of water and dimethyl sulfoxide (DMSO), for solubility. The specific combinations, doses, and solubility solutions used in this study can be found in **Supplementary Table 1**. Each single drug and drug combination were tested in quadruplicate, and each cell line was run in duplicate using a different thawed vial of cells for the replicate. A 384-well plate was used in this experiment where each monotherapy drug or combinations of drugs were run at six distinct doses or combination of doses. Single drugs were plated, and the plates stored at −80°C. The second drug was added on the day of the experiment before the addition of the cells to the entire plate. All drugs were freeze thawed only once. A plate layout can be found in the **Supplementary Material**.

**Table 1:** Mechanism of Action of the Drugs Used.

Drug	Mechanism of Action
Gemcitabine	Ribonucleotide reductase inhibitor
5-Fluorouracil	Thymidylate synthase inhibitor
Epirubicin	Thymidylate synthase inhibitor
Vinorelbine	Microtubule destabilizer
Docetaxel	Microtubule stabilizer
Paclitaxel	Microtubule stabilizer
Oxaliplatin	DNA crosslinker
Carboplatin	DNA crosslinker

Cytotoxic response was measured using the Alamar blue colorimetric assay (BioSource, CA, USA). Plates containing cells and drugs were incubated for 72 h at 37°C, 5% CO_2_ before the addition of the Alamar blue assay. Plates were incubated an additional 24 h before fluorescence was measured using an excitation filter of 535 nm and an emission filter of 595 nm on an Infinite F200 microplate reader with Connect stacker (Tecan Group Ltd) using iControl software (version 1.6). Percent viability was measured relative to appropriate control wells based on mean raw fluorescent units (RFUs).

### Data Quality Control

As described in detail by Brown et al. (2014) a quality control pipeline was used to clean data prior to analysis. The first step in the pipeline consisted of checking each quadruplicate for any deviant values using a coefficient of variation-based approach and replacing deviant values with the mean of the others. For two or more deviant values, the quadruplicates were replaced with the mean across samples. Next, an entire plate viability check was implemented to check for inappropriate mass cell death. This was done by identifying the 90th percentile of RFU values. For a 90th percentile below 2,000, that plate was considered dead and removed from the analysis as the majority of the cells on that plate were dead from something unrelated to drug effect. Third, 10% DMSO negative controls and vehicle controls, contained on each plate, were checked for deviant values as they were used in subsequent steps in the analysis. This was done using a flag and replace iterative algorithm based on a linear regression technique. Deviant values were replaced with those predicted from the linear regression. Raw fluorescent units were then normalized using vehicle and negative controls. Finally, values were checked within each drug in a dose–response regression, again using a flag and replace algorithm, replacing deviant values that may erroneously influence analyses.

### Synergy Quantification

After quality control, the Chou–Talalay combination index (CI) method was used to quantify synergistic interactions between the various drugs tested (Chou and Talalay, 1984). Responses from the Alamar blue assay were run through a quality control pipeline (described in the **Supplementary Material**), and normalized values were used in the CI calculations.

The CI method relies upon the median effects equation of the mass action

$$\frac{F_{a}}{F_{u}}={\left(\frac{D^{}}{D_{m}}\right)}^{m}$$

where *F*_a_ is the fraction of cells affected (i.e., dead), *F*_u_ is the fraction of cells unaffected (i.e., alive), *D* is the dose of drug given, *D*_m_ is the median-effect dose, and *m* is the sigmoidicity of the dose–effect curve. Values for *F*_a_ and *F*_u_ are experimentally determined for an experimentally designed dose *D*. To find values for *D*_m_ and *m*, we can simplify this equation into the following linearized version:

$$\log\left(\frac{F_{a}}{F_{u}}\right)=m*\log D-m*\log D_{m}$$

**Figure 1:**
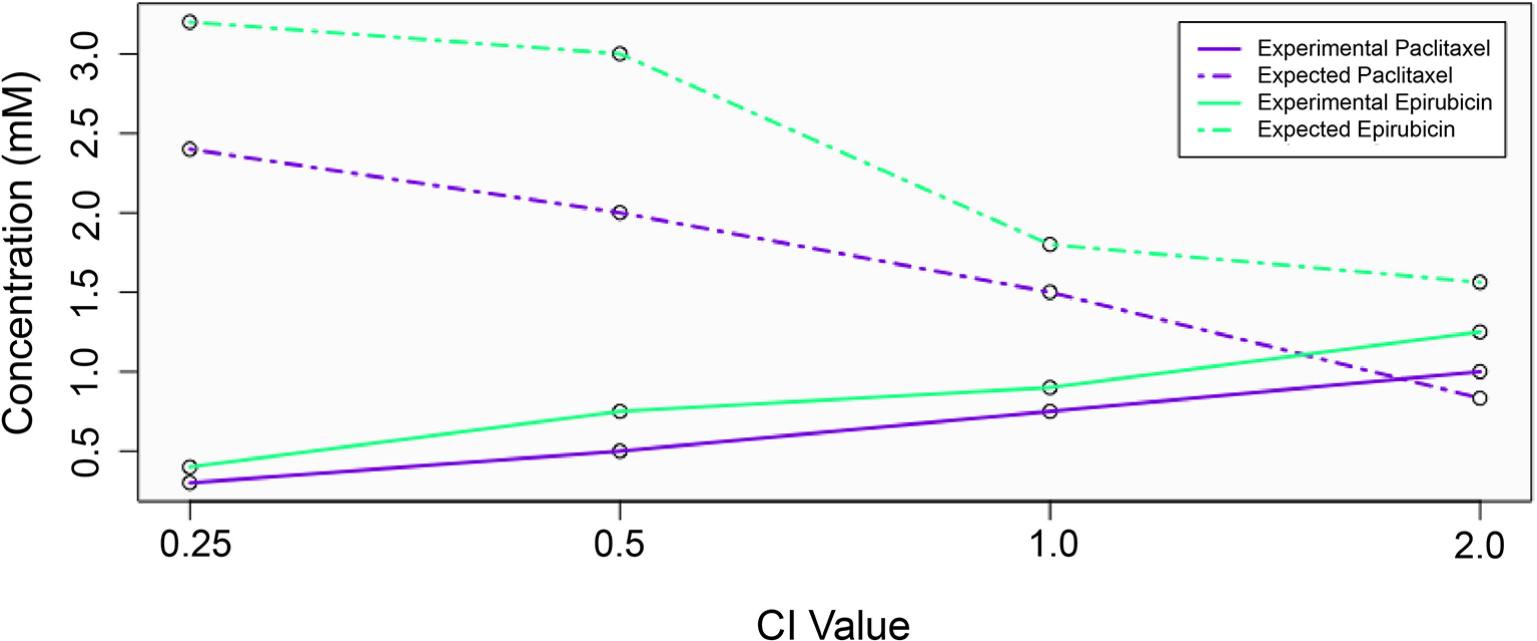
The relationship between expected and experimental drug concentrations and the effect on combination index value. Experimental concentrations for each drug are shown with solid lines, while expected (theoretical) concentrations are shown with dashed lines.

A linear regression is then applied for the various doses (*D*) and responses (*F*_a_/*F*_u_). From this, values for *D*_m_ and *m* can be estimated. These values can then be used to calculate estimates for variables *E*_1_ and *E*_2_ in the following equation giving the CI:

$$CI=\frac{D_{1}}{E_{1}}+\frac{D_{2}}{E_{2}}$$

where *D*_1_ and *D*_2_ are the actual drug doses used in the combinations, and *E*_1_ and *E*_2_ are what individual drug levels would be expected to be needed to achieve the observed response. While *D*_1_ and *D*_2_ are defined experimentally, *E*_1_ and *E*_2_ is calculated using the *D*_m_ and *m* values calculated previously. A CI value less than 1 indicates synergism, greater than 1 indicates antagonism, and equal to 1 indicates additivity. **Figure 1** visually demonstrates the relationship between expected and experimental concentrations (or doses) and corresponding CI values for the example drug combination of epirubicin and paclitaxel. According to Chou (2006), strong synergistic interactions occur below 0.3, and weak interactions are between 0.85 and 1. While values above 1 generally are interpreted as indicating antagonism, larger values do not necessarily indicate increased antagonistic reactions as large values can be erroneously obtained due to poorly fit linear models, and there is no upper bound to the CI value. Consequently, we decided to bound our values at an upper limit of 5.

### Heritability Quantification

Heritability, *H* ^2^, is defined as the proportion of observed variation in a trait attributed to genetic differences among individuals in a population. A particular trait (*P*) can be thought of as a combination of genetic effects (*G*) and environmental effects (*E*). Therefore, the variation in a given trait can be modeled as follows:

Var(P)=Var(G)+Var(E)

Broad sense heritability, *H* ^2^, considers the proportion of all genetic variation (additive effects, dominant effects, etc.) in the total phenotypic variation of a given trait:

$$H^{2}=\frac{Var\left(G\right)}{Var\left(P\right)}$$

We calculated broad sense heritability using Merlin 1.1.2 (MERLIN Software Homepage) for each drug and dose. For the drug combinations, CI values were used as the quantitative trait values in the heritability calculations.

### Genotyping Data

Version 10 of the CEPH database (Cohen et al., 1993; CEPH Genotype Database) was used to download genotype data for each cell line, using error checked markers. Genetic map information was downloaded from the Marshfield database, based on CEPH family genotypes (Broman et al., 1998; Marshfield Genetic Map Mammalian Genotyping Service). Error checking for Mendelian incompatibility, misspecified relationships, and unlikely recombinations was performed as described by Brown et al. (2014). A combined total of 7,815 SNPs and microsatellite markers were used for linkage analysis.

### Linkage Analysis

Linkage analysis is a well-established statistical method for mapping heritable trait genes to their chromosome locations, as reviewed by Bailey-Wilson and Wilson (2011). Genome-wide markers are tested in pedigrees segregating a trait. The statistical method of linkage analysis combines these data to identify chromosome regions likely to harbor genes for the trait. It is based on the observation that genes that reside physically close on a chromosome remain linked during meiosis. For traits for which little is known about the genetic etiology, the identification of the chromosomal location of the gene(s) is the first step in its eventual isolation of the genetic variants that contribute to phenotypic variation. Linkage analysis was conducted to explore any loci contributing to the heritability of the observed responses. Linkage analysis was conducted using the genotype data obtained for each cell line using the Marshfield database as described above. Phenotypes of interest for linkage analysis were defined as cell viability at each drug or drug combination at the dose point that had the highest heritability. For each phenotype, nonparametric linkage analysis was performed using Merlin 1.1.2 (MERLIN Software Homepage) because it is robust to nonnormality in the phenotypic variables of interest. As described in detail by Abecasis et al. (2002), Merlin constructs a likelihood ratio test for linkage based on inheritance vectors (Abecasis et al., 2002).

## Results

### Synergy Within LCLs

**Figure 2:**
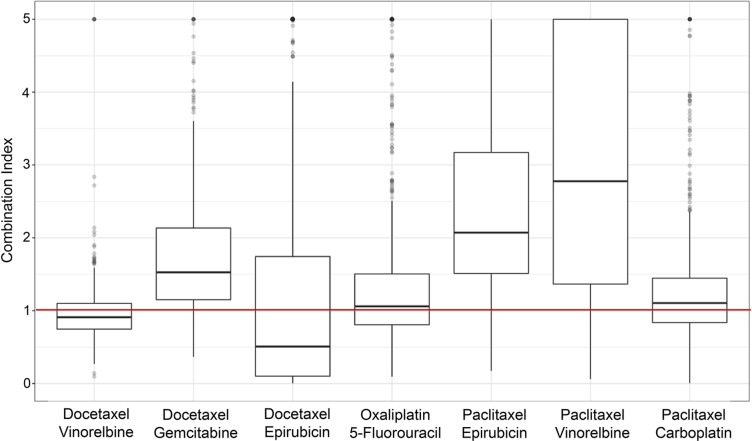
Boxplots of the combination index values (of individual cell lines) across the different drug combinations used in the study. Values below the horizontal, red line indicate synergy, and those above indicate antagonism.

Both synergy and antagonism were observed within the LCLs across varying doses and drug combinations. We saw a range of CI values shown in **Figure 2**. A breakdown of the combination indices for each specific dosage combination in the various drug mixtures can be found in the **Supplementary Table 1**. There is a wide range of values for each of the doses and combinations, with a consistent general pattern that many of the drug combinations showed more synergistic interactions at lower doses.

**Figure 2** shows the seven different drug combinations and their corresponding CI values for all individuals across the entire six doses for all the assayed cell lines. Although certain combinations of specific doses may exhibit synergistic effects, different dose combinations of that same drug mixture may not exhibit any synergism. **Figure 2** shows the overall synergistic effects for a particular drug mixture, across all combinations of doses. As can be seen from the figure, there is a wide range of values, which is necessary for the hypothesis that there is a genetic etiology to the synergistic response.

### Heritability

**Figure 3:**
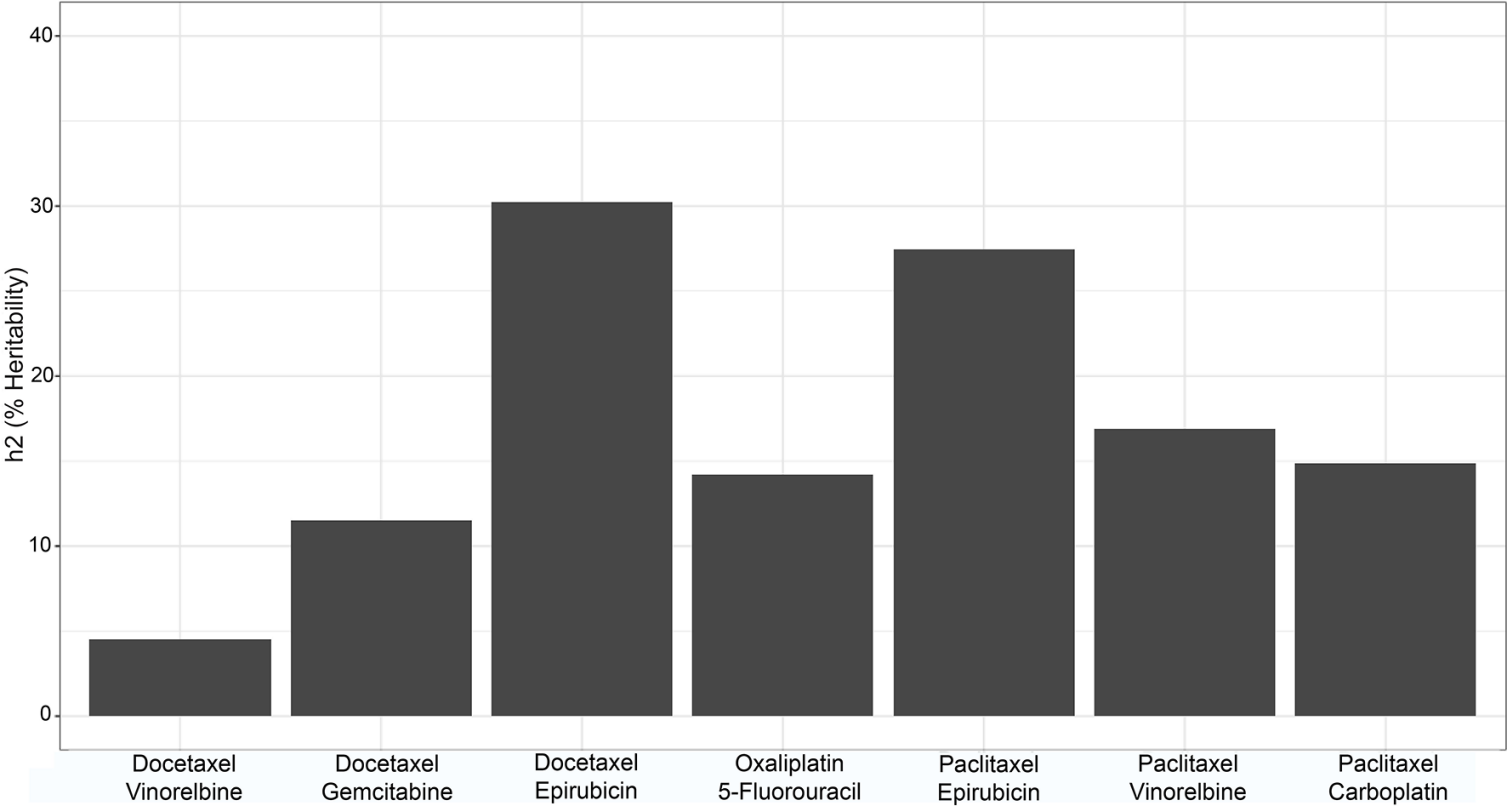
Bar graph of the broad sense heritability of each of the drug combinations.

Heritability for each drug combination at each dose was calculated using Merlin. Broad sense heritability results are shown in **Figure 3**, where the selected heritability value is the largest observed value across doses for each combination. The largest observed heritability values at each dose ranged from around 5% to slightly above 30%, as seen in **Figure 3**.

### Linkage Analysis

**Figure 4:**
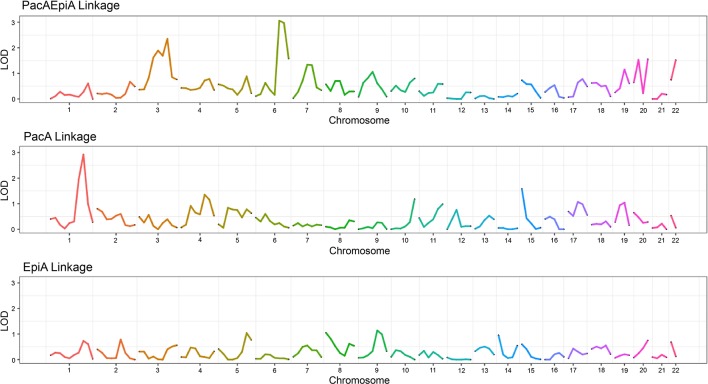
Linkage plot showing the LOD score across chromosomes for two monotherapy drugs as well as their corresponding combination indices.

Linkage analyses were performed for each drug combination and individual drug as previously described. **Figure 4** shows the linkage analysis results for the drug combination of epirubicin and paclitaxel, as well as the monotherapy linkage analyses for each of the drugs used in the combination. As can be seen from the figure, there are some notable differences in the linkage peaks across the three plots. This could indicate that the potential genetic etiology for monotherapy response could be different in comparison to that of combination therapy response. It is interesting to note that there are also some similar, although not as significant, linkage peaks among the three drugs such as those in chromosomes 9, 17, and 19.

**Figure 5:**
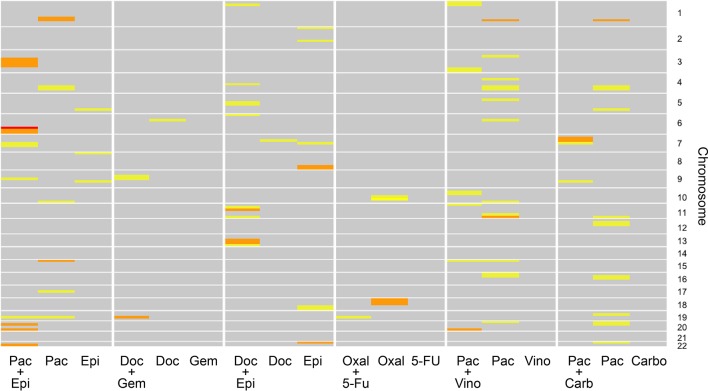
Linkage results across the individual drugs and their combination for each chromosome. The color of each cell representing varying logarithm of odds (LOD) scores where yellow indicates a lower LOD score, and red indicates a large (>3) LOD score. Gray cells indicate a LOD score less than 1.

**Figure 5** graphically describes the various linkage peaks across the numerous combination and monotherapy drugs. Warmer sections of the genome highlighted correspond to higher logarithm of odds (LOD) scores, indicating a region that is inherited along with the dose-response traits. Again, it can be seen that linkage peaks vary between a combination and the corresponding drugs used in monotherapy response across all drug combinations tested.

The data from the combination of epirubicin and paclitaxel produced statistically significant results (LOD > 3.0), so we pulled out genes that are within an LOD ± 1.0 from the top of the peak. The Marshfield database was used to look up marker names for the specific regions with LOD scores > 3. The UCSC Genome Browser (https://genome.ucsc.edu, accessed June 2019) was used to look up all genes in those regions. The results are listed in **Table 2**. Full linkage results for all drug combinations are available in the **Supplementary Table 2**.

**Table 2:** Genes within the LOD > 3 regions identified from the linkage analyses for the paclitaxel epirubicin drug combination. LOD scores above 3 ranged from 3.03 to 3.06.

Chromosome	Gene Names
6	Androgen-induced gene 1 protein (AIG1)
6	AL357146.1
6	AL357146.1
6	AL592429.2
6	FOXO induced long noncoding RNA 1 (FILNC1)
6	Heme-binding protein 2 (HEBP2)
6	Human immunodeficiency virus type I enhancer binding protein 2 (HIVEP2)
6	Neuromedin B receptor (NMBR)

## Discussion

In the current study, we demonstrated that nonadditive effects are common for drug combinations in the LCL system. We demonstrate a range of synergistic and antagonistic interactions across doses. To the best of our knowledge, this is the first time that synergy has been quantified in the LCLs. Our results demonstrate that, with careful experimental design, we can quantify synergy for combination drug response in LCLs. These results support the continued investigation of drug combinations in this model system.

The drug combinations chosen were based on previous studies in cancer cell line and/or tumor tissues. Since the LCL model is arguably a better representation of the “host” genome as opposed to the cancer genome (Jack et al., 2014), it is provocative that nonadditive effects are observed in both the genomes involved in chemotherapy. Our calculated CI values using this LCL model are within the ranges similar to those found for combinations of these same drugs in cancer cell lines (Photiou et al., 1997; Konecny et al., 2001), indicating that the host genome may be of equal importance to the cancer genome in understanding and potentially predicting nonadditivity.

Demonstrating that nonadditive effects are a widespread phenomenon and having shown that both synergy and antagonism exist in these cell lines could have great impacts on testing for nonadditive interactions between a given set of drugs. Repurposing approved drugs within combination therapy has been shown to be more efficient in both time and cost as compared to developing new drugs (Sun et al., 2016). The LCL model can be used in this context.

Importantly, we have shown that the variation in individual synergistic response is a heritable trait. The heritability levels we obtained are consistent with those found in monotherapy drug treatments (Peters et al., 2011). Again, to our knowledge, this has not been shown and has potentially great promise in understanding how synergy and antagonism occur. Specifically, if there is a genetic basis for synergy, this could explain why synergistic responses vary from person to person. It is important to recognize that while these results are still far from clinical relevance, further exploration of these findings could lead to discovery of particular genes for a given combination of drugs that could help explain this variation among synergistic responses in patients. This would be a major contribution to the field of personalized medicine and the manner in which treatments are administered. If the mechanisms of how and why these interactions are occurring are better understood, tailored treatment strategies have the potential for better therapeutic responses with less toxicity. There may be cases where combination therapy is contraindicated. And, again, while the current study is much more preclinical, it has laid the foundation for future research that may be more translational to a clinical setting.

Our linkage experiments demonstrate another provocative result. Linkage analysis was performed both for the single drug responses and the synergistic combination responses. We chose to run a linkage analysis because, while still underpowered, we are using familial based data and do not have an adequate number of samples to perform an association mapping analysis. Running a linkage analysis, however, allows us to potentially narrow down the genetic region associated with the synergy trait. (Bailey-Wilson and Wilson, 2011) Additionally, we were interested in finding these potential genetic regions associated with the synergy response to extend future steps in this process, similar to what has been done in monotherapy drug response genetic analyses. (Jack et al., 2018) Again, while the goal of this analysis was not to discover specific genes involved, a list of genes for the more significant regions (for the paclitaxel–epirubicin drug combination linkage results) has been included in the **Supplementary Material**.

Some of the genes found are annotated as pseudogenes or/and novel transcripts, but several of these putatively linked genes include well-annotated genes with interesting possible biological connections to the drug response outcomes. For example, one of the genes found was the neuromedin B receptor, located on chromosome 6 at base pair location 142,379,467–142,409,936. This gene encodes a 7-transmembrane G protein–coupled receptor that binds neuromedin B, which is a growth factor and mitogen for gastrointestinal epithelial tissue and for normal and neoplastic lung (Corjay et al., 1991; Benya et al., 1995). This receptor has been shown to play a role in in smooth muscle contractions, neuronal responses, and, importantly for this study, the regulation of cell growth (Corjay et al., 1991; Benya et al., 1995). Antagonists of this receptor have recently demonstrated a potential therapeutic use in inhibiting tumor cell growth (Moody et al., 2018). It is provocative that this gene is associated with the synergistic effect of the combination therapy. Further experiments are needed to follow up this interesting finding, along with other genes within this peak.

Overall, the results of the linkage analysis demonstrate that the etiology of synergy may be distinct from that of monotherapy drug response—different genetic regions are linked to the synergistic response than the regions that are linked to the monotherapy response. We recognize that there is limited power in the current dataset, and an important future direction will be to perform replication analysis in a larger dataset. Additionally, it will be necessary to follow up with fine mapping approaches such as genome-wide association analysis. It will also be important to explore how drug-induced gene expression changes differ between monotherapy and combination therapy exposure.

## Data Availability

The datasets generated for this study can be found in Marshfield Database, **Supplementary Figure 1** and **Supplementary Tables 3 and 4**.

## Author Contributions

AM-R, TW, and HM conceived of the experiments. TH performed the cell line experiments. KR, JJ, AM-R, and DR developed and implemented the data analysis plan.

## Funding

This work has been supported by grant 5R01CA161608 from the National Cancer Institute and from intramural support from the National Institute of Environmental Health Sciences.
