# Supplementary material for: Synergistic Chemotherapy Drug Response Is a Genetic Trait in Lymphoblastoid Cell Lines
Source: Front Genet. 2019 Oct 15;10:829. doi: 10.3389/fgene.2019.00829 (PMC6804467; doi:10.3389/fgene.2019.00829)
Supplement: Supplementary file 1 [file Table_1.docx]

Supplementary Material

# Quality Control Pipeline

A quality control (QC) pipeline was implemented to help ensure the legitimacy of the data obtained, as manual error checking with the amount of data is infeasible. Our QC pipeline consists of 5 basic steps:

1. The coefficient of variation is calculated for each of the four replicate relative fluorescence units (RFUs). The point of this is to identify replicates that were too high or low as compared to the others. If the coefficient of variation is above a threshold (.2), the most deviant replicate is removed.
2. Plates that are considered “dead”, having a 90^th^ percentile mean RFU value of less than 1600, are removed from our analysis. This indicates something occurred such as an entire plate contamination or dropped plate, for example, and these data should not be included in the analysis.
3. A “Flag and Replace” (FAR) algorithm is used to identify deviant responses among the vehicles and controls. In our experiment, DMSO was used to increases solubility of specific drugs; these small concentrations of DMSO are referred to as vehicles. Briefly, our FAR algorithm uses a linear regression to identify deviant response values. Responses are deemed as deviant if they are more than a predetermined number of standard deviations away from their corresponding predicted value from a linear regression. Deviant responses are replaced with their predicted responses and this process repeats until convergence. Converges occurs when the average difference of replaced responses between consecutive iterations of the algorithm is below a specific threshold.

Each cell line was run on two separate plates. One plate contained the individual drugs while the second plate contained both individual drugs and drug combinations, the latter shown in Figure 1 below. We use this FAR algorithm to find deviant responses by using controls and vehicles on the same plate as covariates to the linear regression and then by using control and vehicle responses on other plate as covariates to the linear regression.

1. RFUs are normalized based using the positive control and vehicles for each specific drug. As some drugs need DMSO for solubility, this needed to be accounted for when normalizing the responses, as that small amount of DMSO may contribute to cell death. However, we only want to estimate the cell death due to the drug. Additionally, the overall plate viability, estimated by the positive control, is taken into account during normalization.
2. The final step utilizes another FAR algorithm based on the dose-response curve of each drug separately. Covariate to the linear regression used in this FAR algorithm are the normalized responses at each of the other doses within a given drug. If a response is far outside the estimated dose-response curve, based on the predicted value from the linear regression, it is flagged and replaced. Again, this is repeated until convergence.

# Supplementary Figures and Tables

## Supplementary Figure

*Figure* *1.* Plate layout used in the drug combination experiment. Each circle represents 4 wells on a 396 well plate. Full colored circles indicate cells treated with individual drugs at various concentrations. Half circles indicate a combination of drugs (corresponding to the colors used to shade the circle) were used to treat cells.
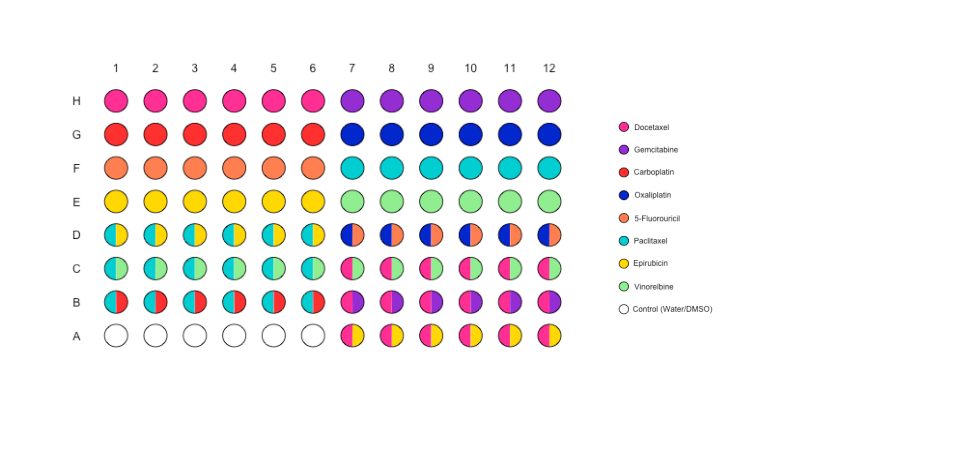


## Supplementary Tables

*Table 1.* Drugs and controls with corresponding doses and concentrations, in milliMolar, used individually.

| Drug | Dose 1 | Dose 2 | Dose 3 | Dose 4 | Dose 5 | Dose 6 |
| --- | --- | --- | --- | --- | --- | --- |
| 5-Fluorouracil | .12911 | .04182 | .01387 | .00617 | .00342 | .000516 |
| Carboplatin | .04193 | .01939 | .011437 | .007041 | .004522 | .001693 |
| Docetaxel | .0001 | .0000782 | .0000635 | .0000516 | .0000419 | .00002 |
| Epirubicin | .00071 | .000288 | .000117 | .000047 | .000019 | .0000078 |
| Gemcitabine | .000188 | .000094 | .000047 | .000024 | .000012 | .000003 |
| Oxaliplatin | .025 | .00631 | .00239 | .00091 | .00034 | .0001 |
| Paclitaxel | .00025 | .000158 | .0001199 | .000091 | .000069 | .000025 |
| Vinorebline | .00012 | .0000657 | .0000534 | .0000433 | .0000352 | .000015 |
| DMSO | 0% | .01% | .1% | 1% | 2% | 10% |

*Table 2.* Drugs combinations and corresponding doses used, reported in milliMolars. For each dose column, the first value corresponds to the first drug listed in combination.

| **Drug Combination** | **Dose 1** | **Dose 2** | **Dose 3** | **Dose 5** | **Dose 5** | **Dose 6** |
| --- | --- | --- | --- | --- | --- | --- |
| Docetaxel + Epirubicin | .0004, .00021 | .0003, .0001 | .0002, .00005 | .0001, .00003 | .00007, .00002 | .00003, .00001 |
| Docetaxel + Gemcitabine | .00026, .0000617 | .00015, .000035 | .00009, .000021 | .00006, .0000134 | .00004, .0000091 | .00002, .0000037 |
| Docetaxel + Vinorelbine | .00015, .00005 | .0001, .00003 | .00008, .00003 | .00006, .00002 | .00005, .00002 | .00002, .00001 |
| Oxaliplatin + 5-Fluorouracil | .012114, .12911 | .003924, .04182 | .001302, .01387 | .000578, .00617 | .00032, .00342 | .0000485, .00052 |
| Paclitaxel + Carboplatin | .0002453, .04193 | .0001134, .01939 | .0000669, .011437 | .0000412, .0070410 | .0000265, .004522 | .0000099, .001693 |
| Paclitaxel + Epirubicin | .0003109, .0002219 | .0001405, .0001002 | .000071, .0000506 | .0000394, .0000281 | .000024, .0000171 | .000009, .0000064 |
| Paclitaxel + Vinorelbine | .0001277, .0000588 | .0000752, .0000347 | .0000590, .0000272 | .0000463, .0000213 | .0000336, .0000167 | .0000144, .0000066 |

*Table 3*. Paclitaxel and Carboplatin combination LOD scores greater than 1 from a linkage analysis performed with Merlin. Marker is the sex averaged position of a specific marker from the Marshfield Comprehensive human genetic maps. Trait indicates which specific dose from Table 2 above, where T1 corresponds to Dose 1, T2 to Dose 2, etc.

| **Chromosome** | **Marker** | **Trait** | **LOD** |
| --- | --- | --- | --- |
| 7 | 36.03 | T5 | 1.23 |
| 7 | 37.26 | T5 | 1.28 |
| 7 | 37.51 | T5 | 1.29 |
| 7 | 38.48 | T5 | 1.35 |
| 7 | 39.02 | T5 | 1.4 |
| 7 | 39.82 | T5 | 1.43 |
| 7 | 40.62 | T5 | 1.38 |
| 7 | 41.16 | T5 | 1.41 |
| 7 | 41.69 | T5 | 1.44 |
| 7 | 43.84 | T5 | 1.55 |
| 7 | 47.08 | T5 | 1.74 |
| 7 | 48.15 | T5 | 1.71 |
| 7 | 48.69 | T5 | 1.6 |
| 7 | 48.84 | T5 | 1.59 |
| 7 | 48.99 | T5 | 1.59 |
| 7 | 49.22 | T5 | 1.58 |
| 7 | 50.29 | T5 | 1.55 |
| 7 | 52.7 | T5 | 1.51 |
| 7 | 53.5 | T5 | 1.5 |
| 7 | 54.11 | T5 | 1.48 |
| 7 | 54.84 | T5 | 1.45 |
| 7 | 57.79 | T5 | 1.27 |
| 7 | 58.86 | T5 | 1.24 |
| 7 | 59.39 | T5 | 1.23 |
| 7 | 59.93 | T5 | 1.21 |
| 7 | 61 | T5 | 1.22 |
| 7 | 61.53 | T5 | 1.23 |
| 7 | 62.07 | T5 | 1.23 |
| 7 | 62.87 | T5 | 1.24 |
| 7 | 63.67 | T5 | 1.24 |
| 7 | 65.55 | T5 | 1.24 |
| 7 | 67.43 | T5 | 1.12 |
| 7 | 67.96 | T5 | 1.12 |
| 7 | 68.5 | T5 | 1.12 |
| 7 | 69.03 | T5 | 1.12 |
| 7 | 69.56 | T5 | 1.12 |
| 7 | 70.64 | T5 | 1.12 |
| 7 | 72.78 | T5 | 1.21 |
| 7 | 73.31 | T5 | 1.22 |
| 7 | 73.84 | T5 | 1.23 |
| 7 | 74.38 | T5 | 1.34 |
| 7 | 74.91 | T5 | 1.33 |
| 7 | 75.44 | T5 | 1.33 |
| 7 | 75.98 | T5 | 1.32 |
| 7 | 76.71 | T5 | 1.31 |
| 7 | 77.91 | T5 | 1.3 |
| 7 | 78.65 | T5 | 1.29 |
| 7 | 79.24 | T5 | 1.28 |
| 7 | 80.42 | T5 | 1.28 |
| 7 | 83.99 | T5 | 1.3 |
| 7 | 84.52 | T5 | 1.31 |
| 7 | 85.32 | T5 | 1.31 |
| 7 | 86.12 | T5 | 1.31 |
| 7 | 87.38 | T5 | 1.31 |
| 7 | 89.88 | T5 | 1.33 |
| 7 | 90.42 | T5 | 1.33 |
| 7 | 90.7 | T5 | 1.34 |
| 7 | 90.95 | T5 | 1.34 |
| 7 | 91.67 | T5 | 1.34 |
| 7 | 93.1 | T5 | 1.34 |
| 7 | 93.63 | T5 | 1.34 |
| 7 | 94.4 | T5 | 1.34 |
| 7 | 95.43 | T5 | 1.34 |
| 7 | 97.38 | T5 | 1.17 |
| 7 | 97.89 | T5 | 1.25 |
| 7 | 98.44 | T5 | 1.25 |
| 7 | 100.05 | T5 | 1.11 |
| 7 | 100.81 | T5 | 1.05 |
| 9 | 109.85 | T5 | 1.03 |

*Table 4*. Paclitaxel and Vinorebline combination LOD scores greater than 1 from a linkage analysis performed with Merlin. Marker is the sex averaged position of a specific marker from the Marshfield Comprehensive human genetic maps. Trait indicates which specific dose from Table 2 above, where T1 corresponds to Dose 1, T2 to Dose 2, etc.

| **Chromosome** | **Marker** | **Trait** | **LOD** |
| --- | --- | --- | --- |
| 1 | 23.35 | T4 | 1.04 |
| 1 | 24.68 | T4 | 1.08 |
| 1 | 29.93 | T4 | 1.25 |
| 1 | 31.02 | T4 | 1.25 |
| 1 | 33.75 | T4 | 1.27 |
| 1 | 35.4 | T4 | 1.28 |
| 1 | 37.05 | T4 | 1.28 |
| 1 | 38.51 | T4 | 1.28 |
| 1 | 41.92 | T4 | 1.19 |
| 1 | 43.72 | T4 | 1.15 |
| 1 | 45.33 | T4 | 1.1 |
| 1 | 46.61 | T4 | 1.07 |
| 1 | 48.53 | T4 | 1.02 |
| 1 | 49.07 | T4 | 1 |
| 3 | 191.79 | T4 | 1 |
| 3 | 192.85 | T4 | 1.03 |
| 3 | 193.75 | T4 | 1.05 |
| 3 | 194.68 | T4 | 1.07 |
| 3 | 195.6 | T4 | 1.09 |
| 3 | 198.68 | T4 | 1.15 |
| 3 | 201.14 | T4 | 1.18 |
| 3 | 203.28 | T4 | 1.19 |
| 3 | 203.81 | T4 | 1.19 |
| 3 | 205.56 | T4 | 1.19 |
| 3 | 206.43 | T4 | 1.18 |
| 3 | 207.73 | T4 | 1.18 |
| 3 | 209.41 | T4 | 1.18 |
| 3 | 210.09 | T4 | 1.16 |
| 3 | 212.61 | T4 | 1.06 |
| 3 | 213.64 | T4 | 1.02 |
| 10 | 33.18 | T1 | 1 |
| 10 | 33.48 | T1 | 1.01 |
| 10 | 35.41 | T1 | 1.08 |
| 10 | 36.06 | T1 | 1.1 |
| 10 | 37.9 | T1 | 1.17 |
| 10 | 40.36 | T1 | 1.26 |
| 10 | 41.07 | T1 | 1.28 |
| 10 | 41.79 | T1 | 1.31 |
| 10 | 42.5 | T1 | 1.32 |
| 10 | 45.7 | T1 | 1.37 |
| 10 | 46.23 | T1 | 1.38 |
| 10 | 46.94 | T1 | 1.41 |
| 10 | 48.36 | T1 | 1.46 |
| 10 | 48.9 | T1 | 1.43 |
| 10 | 49.43 | T1 | 1.39 |
| 10 | 52.1 | T1 | 1.24 |
| 10 | 54.23 | T1 | 1.11 |
| 10 | 56.89 | T1 | 1.07 |
| 10 | 57.42 | T1 | 1.06 |
| 10 | 57.96 | T1 | 1.05 |
| 10 | 59.03 | T1 | 1.04 |
| 10 | 60.64 | T1 | 1.01 |
| 11 | 0 | T2 | 1.18 |
| 11 | 2.11 | T2 | 1.42 |
| 11 | 2.79 | T2 | 1.35 |
| 11 | 4.84 | T2 | 1.2 |
| 11 | 16.12 | T2 | 1 |
| 11 | 17.19 | T2 | 1 |
| 15 | 4.78 | T4 | 1.09 |
| 15 | 6.11 | T4 | 1.15 |
| 15 | 6.92 | T4 | 1.18 |
| 15 | 9.85 | T4 | 1.3 |
| 15 | 12.3 | T4 | 1.16 |
| 15 | 13.06 | T4 | 1.12 |
| 15 | 14.58 | T4 | 1.03 |
| 20 | 83.51 | T2 | 1.13 |
| 20 | 84.23 | T2 | 1.72 |
| 20 | 84.78 | T2 | 1.83 |
| 20 | 85.41 | T2 | 1.87 |
| 20 | 86.98 | T2 | 1.86 |
| 20 | 90.06 | T2 | 1.61 |
| 20 | 90.07 | T2 | 1.6 |

*Table 5*. Paclitaxel and Epirubicin combination LOD scores greater than 1 from a linkage analysis performed with Merlin. Marker is the sex averaged position of a specific marker from the Marshfield Comprehensive human genetic maps. Trait indicates which specific dose from Table 2 above, where T1 corresponds to Dose 1, T2 to Dose 2, etc.

| **Chromosome** | **Marker** | **Trait** | **LOD** |
| --- | --- | --- | --- |
| 3 | 89.91 | T3 | 1 |
| 3 | 91.18 | T3 | 1.09 |
| 3 | 94.99 | T3 | 1.36 |
| 3 | 96.12 | T3 | 1.44 |
| 3 | 96.66 | T3 | 1.47 |
| 3 | 97.75 | T3 | 1.53 |
| 3 | 99.38 | T3 | 1.62 |
| 3 | 101.55 | T3 | 1.71 |
| 3 | 102.64 | T3 | 1.75 |
| 3 | 103.72 | T3 | 1.79 |
| 3 | 104.83 | T3 | 1.81 |
| 3 | 107.19 | T3 | 1.86 |
| 3 | 108.48 | T3 | 1.87 |
| 3 | 109.22 | T3 | 1.88 |
| 3 | 110.82 | T3 | 1.89 |
| 3 | 111.89 | T3 | 1.82 |
| 3 | 112.42 | T3 | 1.8 |
| 3 | 112.96 | T3 | 1.78 |
| 3 | 113.58 | T3 | 1.76 |
| 3 | 114.02 | T3 | 1.74 |
| 3 | 114.56 | T3 | 1.75 |
| 3 | 115.09 | T3 | 1.75 |
| 3 | 115.76 | T3 | 1.75 |
| 3 | 117.76 | T3 | 1.75 |
| 3 | 119.09 | T3 | 1.75 |
| 3 | 120.43 | T3 | 1.74 |
| 3 | 121.67 | T3 | 1.74 |
| 3 | 124.16 | T3 | 1.72 |
| 3 | 124.83 | T3 | 1.71 |
| 3 | 126.83 | T3 | 1.69 |
| 3 | 127.89 | T3 | 1.67 |
| 3 | 129.11 | T3 | 1.66 |
| 3 | 129.73 | T3 | 1.65 |
| 3 | 131.83 | T3 | 1.62 |
| 3 | 133.93 | T3 | 1.59 |
| 3 | 134.64 | T3 | 1.57 |
| 3 | 136.32 | T3 | 1.55 |
| 3 | 137.44 | T3 | 1.53 |
| 3 | 138 | T3 | 1.52 |
| 3 | 139.12 | T3 | 1.5 |
| 3 | 139.24 | T3 | 1.5 |
| 3 | 139.45 | T3 | 1.5 |
| 3 | 139.65 | T3 | 1.5 |
| 3 | 140.19 | T3 | 1.49 |
| 3 | 141.79 | T3 | 1.49 |
| 3 | 143.94 | T3 | 1.47 |
| 3 | 146.6 | T3 | 1.4 |
| 3 | 148.2 | T3 | 1.28 |
| 3 | 149.26 | T3 | 1.18 |
| 3 | 149.8 | T3 | 1.13 |
| 3 | 149.97 | T3 | 1.11 |
| 3 | 150.64 | T3 | 1.04 |
| 3 | 153.18 | T3 | 1 |
| 3 | 153.74 | T3 | 1.14 |
| 3 | 154.48 | T3 | 1.31 |
| 3 | 158.38 | T3 | 2.06 |
| 3 | 159.8 | T3 | 2.24 |
| 3 | 161.04 | T3 | 2.35 |
| 3 | 163.18 | T3 | 2.19 |
| 3 | 164.25 | T3 | 2.08 |
| 3 | 165.32 | T3 | 1.94 |
| 3 | 165.85 | T3 | 1.87 |
| 3 | 166.93 | T3 | 1.7 |
| 3 | 168.94 | T3 | 1.36 |
| 3 | 169.6 | T3 | 1.24 |
| 3 | 170.14 | T3 | 1.14 |
| 3 | 170.67 | T3 | 1.04 |
| 3 | 149.26 | T4 | 1.13 |
| 3 | 149.8 | T4 | 1.19 |
| 3 | 149.97 | T4 | 1.21 |
| 3 | 150.64 | T4 | 1.28 |
| 3 | 151.49 | T4 | 1.35 |
| 3 | 152.62 | T4 | 1.28 |
| 3 | 153.18 | T4 | 1.24 |
| 3 | 153.74 | T4 | 1.19 |
| 3 | 154.48 | T4 | 1.13 |
| 6 | 135.47 | T3 | 1.39 |
| 6 | 136.97 | T3 | 1.8 |
| 6 | 137.74 | T3 | 2 |
| 6 | 141.15 | T3 | 2.74 |
| 6 | 142.86 | T3 | 3.06 |
| 6 | 143.4 | T3 | 3.03 |
| 6 | 144.46 | T3 | 2.97 |
| 6 | 146.06 | T3 | 2.88 |
| 6 | 146.6 | T3 | 2.84 |
| 6 | 147.13 | T3 | 2.8 |
| 6 | 149.13 | T3 | 2.68 |
| 6 | 149.8 | T3 | 2.64 |
| 6 | 151.42 | T3 | 2.56 |
| 6 | 153.04 | T3 | 2.5 |
| 6 | 153.57 | T3 | 2.48 |
| 6 | 154.1 | T3 | 2.45 |
| 6 | 154.64 | T3 | 2.43 |
| 6 | 155.17 | T3 | 2.4 |
| 6 | 156.24 | T3 | 2.36 |
| 6 | 156.77 | T3 | 2.34 |
| 6 | 157.31 | T3 | 2.32 |
| 6 | 157.84 | T3 | 2.29 |
| 6 | 159.44 | T3 | 2.2 |
| 6 | 159.98 | T3 | 2.17 |
| 6 | 161.55 | T3 | 2.12 |
| 6 | 161.59 | T3 | 2.11 |
| 6 | 164.78 | T3 | 1.9 |
| 6 | 166.39 | T3 | 1.79 |
| 6 | 167.78 | T3 | 1.71 |
| 6 | 169.95 | T3 | 1.58 |
| 6 | 173.31 | T3 | 1.39 |
| 6 | 177.88 | T3 | 1.16 |
| 6 | 179.07 | T3 | 1.11 |
| 7 | 86.12 | T3 | 1.01 |
| 7 | 87.38 | T3 | 1.07 |
| 7 | 89.88 | T3 | 1.18 |
| 7 | 90.42 | T3 | 1.2 |
| 7 | 90.7 | T3 | 1.21 |
| 7 | 90.95 | T3 | 1.22 |
| 7 | 91.67 | T3 | 1.25 |
| 7 | 93.1 | T3 | 1.29 |
| 7 | 93.63 | T3 | 1.31 |
| 7 | 94.4 | T3 | 1.32 |
| 7 | 95.43 | T3 | 1.34 |
| 7 | 97.38 | T3 | 1.26 |
| 7 | 97.89 | T3 | 1.24 |
| 7 | 98.44 | T3 | 1.2 |
| 7 | 100.05 | T3 | 1.15 |
| 7 | 100.81 | T3 | 1.12 |
| 7 | 103.63 | T3 | 1.03 |
| 7 | 108.59 | T3 | 1.16 |
| 7 | 109.12 | T3 | 1.21 |
| 7 | 109.66 | T3 | 1.24 |
| 7 | 111.26 | T3 | 1.29 |
| 7 | 111.79 | T3 | 1.3 |
| 7 | 112.1 | T3 | 1.31 |
| 7 | 112.32 | T3 | 1.31 |
| 7 | 112.85 | T3 | 1.32 |
| 7 | 113.39 | T3 | 1.32 |
| 7 | 113.92 | T3 | 1.28 |
| 7 | 115.96 | T3 | 1.22 |
| 7 | 118.9 | T3 | 1.08 |
| 7 | 119.81 | T3 | 1.03 |
| 9 | 79.06 | T3 | 1.03 |
| 9 | 79.77 | T3 | 1.05 |
| 9 | 80.31 | T3 | 1.06 |
| 19 | 58.69 | T4 | 1.01 |
| 19 | 59.36 | T4 | 1.02 |
| 19 | 61.49 | T4 | 1.15 |
| 19 | 62.03 | T4 | 1.09 |
| 19 | 62.25 | T4 | 1.05 |
| 20 | 83.51 | T3 | 1.05 |
| 20 | 84.23 | T3 | 1.38 |
| 20 | 84.78 | T3 | 1.55 |
| 20 | 85.41 | T3 | 1.44 |
| 20 | 86.98 | T3 | 1.13 |
| 20 | 29.09 | T4 | 1.03 |
| 20 | 30.56 | T4 | 1.17 |
| 20 | 31.43 | T4 | 1.25 |
| 20 | 32.3 | T4 | 1.32 |
| 20 | 32.94 | T4 | 1.36 |
| 20 | 34.22 | T4 | 1.41 |
| 20 | 35.51 | T4 | 1.49 |
| 20 | 36.58 | T4 | 1.52 |
| 20 | 37.65 | T4 | 1.53 |
| 20 | 38.72 | T4 | 1.12 |
| 22 | 31.3 | T3 | 1.22 |
| 22 | 31.84 | T3 | 1.37 |
| 22 | 32.39 | T3 | 1.52 |
| 22 | 32.44 | T3 | 1.52 |
| 22 | 32.51 | T3 | 1.52 |
| 22 | 32.93 | T3 | 1.5 |
| 22 | 33.76 | T3 | 1.47 |
| 22 | 36.22 | T3 | 1.38 |
| 22 | 37.82 | T3 | 1.31 |
| 22 | 38.62 | T3 | 1.27 |
| 22 | 41.42 | T3 | 1.15 |
| 22 | 42.81 | T3 | 1.08 |
| 22 | 44.32 | T3 | 1.04 |
| 22 | 45.82 | T3 | 1.02 |
| 22 | 46.42 | T3 | 1.01 |
| 22 | 47.31 | T3 | 1 |

*Table 6.*  Oxaliplatin and 5-Fluoruracil combination LOD scores greater than 1 from a linkage analysis performed with Merlin. Marker is the sex averaged position of a specific marker from the Marshfield Comprehensive human genetic maps. Trait indicates which specific dose from Table 2 above, where T1 corresponds to Dose 1, T2 to Dose 2, etc.

| **Chromosome** | **Marker** | **Trait** | **LOD** |
| --- | --- | --- | --- |
| 19 | 74.07 | T5 | 1 |
| 19 | 74.77 | T5 | 1 |
| 19 | 75.41 | T5 | 1 |

*Table 7*. Docetaxel and Epirubicin combination LOD scores greater than 1 from a linkage analysis performed with Merlin. Marker is the sex averaged position of a specific marker from the Marshfield Comprehensive human genetic maps. Trait indicates which specific dose from Table 2 above, where T1 corresponds to Dose 1, T2 to Dose 2, etc.

| **Chromosome** | **Marker** | **Trait** | **LOD** |
| --- | --- | --- | --- |
| 1 | 29.93 | T2 | 1.14 |
| 1 | 31.02 | T2 | 1.18 |
| 1 | 33.75 | T2 | 1.28 |
| 1 | 35.4 | T2 | 1.35 |
| 1 | 37.05 | T2 | 1.42 |
| 1 | 38.51 | T2 | 1.46 |
| 1 | 41.92 | T2 | 1.46 |
| 1 | 43.72 | T2 | 1.46 |
| 1 | 45.33 | T2 | 1.45 |
| 1 | 46.61 | T2 | 1.44 |
| 1 | 48.53 | T2 | 1.41 |
| 1 | 49.07 | T2 | 1.33 |
| 1 | 50.28 | T2 | 1.12 |
| 4 | 106.89 | T6 | 1 |
| 4 | 107.95 | T6 | 1.01 |
| 4 | 109.02 | T6 | 1.03 |
| 4 | 112.62 | T6 | 1.08 |
| 4 | 114.04 | T6 | 1.1 |
| 4 | 114.67 | T6 | 1.1 |
| 4 | 116.37 | T6 | 1.13 |
| 4 | 117.06 | T6 | 1.14 |
| 5 | 94.8 | T1 | 1.01 |
| 5 | 94.91 | T1 | 1.03 |
| 5 | 95.09 | T1 | 1.03 |
| 5 | 95.25 | T1 | 1.03 |
| 5 | 95.4 | T1 | 1.04 |
| 5 | 97.21 | T1 | 1.07 |
| 5 | 97.82 | T1 | 1.08 |
| 5 | 99.42 | T1 | 1.1 |
| 5 | 101.02 | T1 | 1.1 |
| 5 | 102.62 | T1 | 1.1 |
| 5 | 104.76 | T1 | 1.08 |
| 5 | 105.29 | T1 | 1.07 |
| 6 | 7.02 | T5 | 1.08 |
| 6 | 9.18 | T5 | 1.15 |
| 11 | 30.88 | T2 | 1.05 |
| 11 | 32.29 | T2 | 1.08 |
| 11 | 32.63 | T2 | 1.09 |
| 11 | 33.02 | T2 | 1.1 |
| 11 | 33.57 | T2 | 1.13 |
| 11 | 35.21 | T2 | 1.19 |
| 11 | 35.76 | T2 | 1.2 |
| 11 | 37.62 | T2 | 1.19 |
| 11 | 39.18 | T2 | 1.14 |
| 11 | 40.12 | T2 | 1.09 |
| 11 | 40.9 | T2 | 1.04 |
| 11 | 40.95 | T2 | 1.03 |
| 11 | 42.55 | T2 | 1.03 |
| 11 | 43.16 | T2 | 1.03 |
| 11 | 45.94 | T2 | 1.02 |
| 11 | 47.06 | T2 | 1.01 |
| 11 | 47.61 | T2 | 1.01 |
| 11 | 48.73 | T2 | 1 |
| 11 | 51.95 | T2 | 1.51 |
| 11 | 53.02 | T2 | 1.49 |
| 11 | 53.56 | T2 | 1.47 |
| 11 | 53.87 | T2 | 1.47 |
| 11 | 54.09 | T2 | 1.46 |
| 11 | 127.33 | T2 | 1.07 |
| 13 | 52.64 | T1 | 1 |
| 13 | 53.17 | T1 | 1.03 |
| 13 | 55.31 | T1 | 1.1 |
| 13 | 55.85 | T1 | 1.11 |
| 13 | 56.38 | T1 | 1.12 |
| 13 | 57.46 | T1 | 1.12 |
| 13 | 58.54 | T1 | 1.14 |
| 13 | 59.07 | T1 | 1.15 |
| 13 | 59.79 | T1 | 1.17 |
| 13 | 61.17 | T1 | 1.18 |
| 13 | 62.83 | T1 | 1.19 |
| 13 | 63.9 | T1 | 1.19 |
| 13 | 64.43 | T1 | 1.2 |
| 13 | 64.97 | T1 | 1.23 |
| 13 | 66.04 | T1 | 1.42 |
| 13 | 66.59 | T1 | 1.5 |
| 13 | 67.12 | T1 | 1.55 |
| 13 | 67.65 | T1 | 1.6 |
| 13 | 68.19 | T1 | 1.62 |
| 13 | 68.44 | T1 | 1.63 |
| 13 | 68.73 | T1 | 1.63 |
| 13 | 70.13 | T1 | 1.58 |
| 13 | 71.06 | T1 | 1.5 |
| 13 | 72.05 | T1 | 1.4 |
| 13 | 73.04 | T1 | 1.29 |
| 13 | 75.19 | T1 | 1.36 |
| 13 | 76.26 | T1 | 1.4 |
| 13 | 76.31 | T1 | 1.4 |
| 13 | 76.66 | T1 | 1.41 |
| 13 | 76.8 | T1 | 1.41 |
| 13 | 77.47 | T1 | 1.4 |
| 13 | 79.49 | T1 | 1.36 |
| 13 | 81.64 | T1 | 1.34 |
| 13 | 82.93 | T1 | 1.32 |
| 13 | 83.57 | T1 | 1.31 |
| 13 | 84.87 | T1 | 1.31 |
| 13 | 85.41 | T1 | 1.31 |
| 13 | 87.03 | T1 | 1.29 |
| 13 | 90.27 | T1 | 1.18 |
| 13 | 93.52 | T1 | 1 |

*Table 8*. Docetaxel and Gemcitabine combination LOD scores greater than 1 from a linkage analysis performed with Merlin. Marker is the sex averaged position of a specific marker from the Marshfield Comprehensive human genetic maps. Trait indicates which specific dose from Table 2 above, where T1 corresponds to Dose 1, T2 to Dose 2, etc.

| **Chromosome** | **Marker** | **Trait** | **LOD** |
| --- | --- | --- | --- |
| 9 | 63.65 | T5 | 1.06 |
| 9 | 64.72 | T5 | 1.11 |
| 9 | 65.79 | T5 | 1.15 |
| 9 | 66.32 | T5 | 1.17 |
| 9 | 66.86 | T5 | 1.2 |
| 9 | 67.39 | T5 | 1.22 |
| 9 | 67.93 | T5 | 1.24 |
| 9 | 70.33 | T5 | 1.1 |
| 19 | 58.69 | T6 | 2.13 |
| 19 | 59.36 | T6 | 1.36 |

*Table 9*. Docetaxel and Vinorebine combination LOD scores greater than 1 from a linkage analysis performed with Merlin. Marker is the sex averaged position of a specific marker from the Marshfield Comprehensive human genetic maps. Trait indicates which specific dose from Table 2 above, where T1 corresponds to Dose 1, T2 to Dose 2, etc.

| **Chromosome** | **Marker** | **Trait** | **LOD** |
| --- | --- | --- | --- |
| 6 | 80.99 | T2 | 1.01 |
| 6 | 81.52 | T2 | 1.18 |
| 6 | 82.59 | T2 | 1.33 |
| 6 | 83.26 | T2 | 1.2 |
| 6 | 84.15 | T2 | 1.19 |
| 6 | 87.29 | T2 | 1.35 |
| 6 | 88.63 | T2 | 1.5 |
| 6 | 89.23 | T2 | 1.59 |
| 6 | 89.83 | T2 | 1.69 |
| 6 | 90.43 | T2 | 1.8 |
| 6 | 91.34 | T2 | 1.48 |
| 6 | 92.25 | T2 | 1.15 |
| 6 | 81.52 | T3 | 1.04 |
| 7 | 98.44 | T4 | 2.06 |
| 7 | 100.05 | T4 | 1.5 |
| 7 | 100.81 | T4 | 1.32 |
| 7 | 149.9 | T4 | 1.3 |
| 7 | 151.25 | T4 | 1.21 |
| 7 | 155.1 | T4 | 1.14 |
| 7 | 161.21 | T4 | 1.52 |
| 11 | 89.69 | T3 | 1.06 |
| 11 | 30.88 | T4 | 1.29 |
| 11 | 32.29 | T4 | 1.6 |
| 11 | 32.63 | T4 | 1.61 |
| 11 | 33.02 | T4 | 1.56 |
| 11 | 33.57 | T4 | 1.65 |
| 11 | 35.21 | T4 | 1.62 |
| 11 | 35.76 | T4 | 1.57 |
| 11 | 37.62 | T4 | 1.37 |
| 11 | 39.18 | T4 | 1.26 |
| 11 | 40.12 | T4 | 1.14 |
| 11 | 42.55 | T4 | 1.19 |
| 11 | 43.16 | T4 | 1.25 |
| 11 | 45.94 | T4 | 1.36 |
| 11 | 47.06 | T4 | 1.4 |
| 11 | 47.61 | T4 | 1.45 |
| 11 | 48.73 | T4 | 1.63 |
| 11 | 49.73 | T4 | 1.47 |
| 11 | 50.88 | T4 | 1.36 |
| 11 | 51.42 | T4 | 1.32 |
| 11 | 51.95 | T4 | 1.55 |
| 11 | 53.02 | T4 | 1.5 |
| 11 | 53.56 | T4 | 1.49 |
| 11 | 53.87 | T4 | 1.48 |
| 11 | 54.09 | T4 | 1.48 |
| 11 | 54.75 | T4 | 1.47 |
| 11 | 56.76 | T4 | 1.5 |
| 11 | 57.35 | T4 | 1.53 |
| 11 | 57.83 | T4 | 1.57 |
| 11 | 58.4 | T4 | 1.61 |
| 11 | 59.24 | T4 | 1.59 |
| 11 | 59.77 | T4 | 1.61 |
| 11 | 60.09 | T4 | 1.62 |
| 11 | 61.78 | T4 | 1.66 |
| 11 | 62.62 | T4 | 1.68 |
| 11 | 65.05 | T4 | 1.72 |
| 11 | 67.48 | T4 | 1.77 |
| 11 | 68.01 | T4 | 1.78 |
| 11 | 68.55 | T4 | 1.79 |
| 11 | 71.6 | T4 | 1.91 |
| 11 | 72.82 | T4 | 1.69 |
| 11 | 73.64 | T4 | 1.64 |
| 11 | 75.3 | T4 | 1.63 |
| 11 | 76.13 | T4 | 1.65 |
| 11 | 77.78 | T4 | 1.72 |
| 11 | 79.43 | T4 | 1.52 |
| 11 | 79.98 | T4 | 1.56 |
| 11 | 81.26 | T4 | 1.64 |
| 11 | 82.53 | T4 | 1.69 |
| 11 | 82.57 | T4 | 1.69 |
| 11 | 83.83 | T4 | 1.66 |
| 11 | 84.13 | T4 | 1.64 |
| 11 | 84.31 | T4 | 1.62 |
| 11 | 84.38 | T4 | 1.62 |
| 11 | 85.48 | T4 | 1.43 |
| 11 | 86.08 | T4 | 1.47 |
| 11 | 86.98 | T4 | 1.28 |
| 11 | 87.89 | T4 | 1.16 |
| 11 | 88.49 | T4 | 1 |
| 11 | 89.69 | T4 | 1.11 |
| 11 | 93.12 | T4 | 1.28 |
| 11 | 94.18 | T4 | 1.29 |
| 11 | 94.72 | T4 | 1.22 |
| 11 | 95.43 | T4 | 1.13 |
| 11 | 96.85 | T4 | 1.04 |
| 11 | 97.39 | T4 | 1.04 |
| 11 | 97.92 | T4 | 1.07 |
| 11 | 104.03 | T4 | 1.25 |
| 11 | 104.6 | T4 | 1.4 |
| 11 | 105.17 | T4 | 1.36 |
| 11 | 105.74 | T4 | 1.26 |
| 11 | 106.31 | T4 | 1.35 |
| 11 | 107.45 | T4 | 1.26 |
| 11 | 108.59 | T4 | 1.29 |
| 11 | 110.73 | T4 | 2.18 |
| 11 | 112.33 | T4 | 2.68 |
| 11 | 112.87 | T4 | 2.62 |
| 11 | 113.13 | T4 | 2.56 |
| 11 | 113.4 | T4 | 2.43 |
| 11 | 113.93 | T4 | 1.6 |
| 11 | 115.53 | T4 | 1.18 |
| 11 | 116.07 | T4 | 1.04 |
| 11 | 2.11 | T5 | 1.57 |
| 11 | 2.79 | T5 | 1.39 |
| 11 | 32.29 | T5 | 1.09 |
| 11 | 32.63 | T5 | 1.17 |
| 11 | 33.02 | T5 | 1.24 |
| 11 | 33.57 | T5 | 1.22 |
| 11 | 35.21 | T5 | 1.03 |
| 11 | 40.9 | T5 | 1.15 |
| 11 | 40.95 | T5 | 1.18 |
| 11 | 42.55 | T5 | 1.31 |
| 11 | 43.16 | T5 | 1.23 |
| 11 | 53.02 | T5 | 1.02 |
| 11 | 53.56 | T5 | 1.04 |
| 11 | 53.87 | T5 | 1.06 |
| 11 | 54.09 | T5 | 1.08 |
| 11 | 54.75 | T5 | 1.22 |
| 11 | 56.76 | T5 | 1.53 |
| 11 | 57.35 | T5 | 1.68 |
| 11 | 57.83 | T5 | 1.82 |
| 11 | 58.4 | T5 | 1.92 |
| 11 | 59.24 | T5 | 1.91 |
| 11 | 59.77 | T5 | 1.88 |
| 11 | 60.09 | T5 | 1.87 |
| 11 | 61.78 | T5 | 1.78 |
| 11 | 62.62 | T5 | 1.74 |
| 11 | 65.05 | T5 | 1.69 |
| 11 | 67.48 | T5 | 1.7 |
| 11 | 68.01 | T5 | 1.71 |
| 11 | 68.55 | T5 | 1.73 |
| 11 | 71.6 | T5 | 1.89 |
| 11 | 72.82 | T5 | 1.61 |
| 11 | 73.64 | T5 | 1.54 |
| 11 | 75.3 | T5 | 1.5 |
| 11 | 76.13 | T5 | 1.51 |
| 11 | 77.78 | T5 | 1.59 |
| 11 | 79.43 | T5 | 1.56 |
| 11 | 79.98 | T5 | 1.61 |
| 11 | 81.26 | T5 | 1.76 |
| 11 | 82.53 | T5 | 1.91 |
| 11 | 82.57 | T5 | 1.92 |
| 11 | 83.83 | T5 | 2.03 |
| 11 | 84.13 | T5 | 2.04 |
| 11 | 84.31 | T5 | 2.04 |
| 11 | 84.38 | T5 | 2.04 |
| 11 | 85.48 | T5 | 1.92 |
| 11 | 86.08 | T5 | 1.26 |
| 20 | 84.78 | T4 | 1.18 |
| 20 | 85.41 | T4 | 1.13 |
| 20 | 84.78 | T5 | 1.05 |
